# A substrate-bound structure of cyanobacterial biliverdin reductase identifies stacked substrates as critical for activity

**DOI:** 10.1038/ncomms14397

**Published:** 2017-02-07

**Authors:** Haruna Takao, Kei Hirabayashi, Yuki Nishigaya, Haruna Kouriki, Tetsuko Nakaniwa, Yoshinori Hagiwara, Jiro Harada, Hideaki Sato, Toshimasa Yamazaki, Yoichi Sakakibara, Masahito Suiko, Yujiro Asada, Yasuhiro Takahashi, Ken Yamamoto, Keiichi Fukuyama, Masakazu Sugishima, Kei Wada

**Affiliations:** 1 Organization for Promotion of Tenure Track, University of Miyazaki, Miyazaki 889-1692, Japan; 2 Graduate School of Medicine and Veterinary Medicine, University of Miyazaki, Miyazaki 889-1692, Japan; 3 Advanced Analysis Center, National Agriculture and Food Research Organization, Ibaraki 305-8602, Japan; 4 Department of Biological Sciences, Graduate School of Science, Osaka University, Osaka 560-0043, Japan; 5 Department of Biochemistry and Applied Chemistry, National Institute of Technology, Kurume College, Fukuoka 830-8555, Japan; 6 Department of Medical Biochemistry, Kurume University School of Medicine, Fukuoka 830-0011, Japan; 7 Department of Biochemistry and Applied Biosciences, Faculty of Agriculture, University of Miyazaki, Miyazaki 889-2192, Japan; 8 Department of Pathology, Faculty of Medicine, University of Miyazaki, Miyazaki 889-1692, Japan; 9 Division of Life Science, Graduate School of Science and Engineering, Saitama University, Saitama 338-8570, Japan; 10 Department of Applied Chemistry, Graduate School of Engineering, Osaka University, Osaka 565-0871, Japan

## Abstract

Biliverdin reductase catalyses the last step in haem degradation and produces the major lipophilic antioxidant bilirubin via reduction of biliverdin, using NAD(P)H as a cofactor. Despite the importance of biliverdin reductase in maintaining the redox balance, the molecular details of the reaction it catalyses remain unknown. Here we present the crystal structure of biliverdin reductase in complex with biliverdin and NADP^+^. Unexpectedly, two biliverdin molecules, which we designated the proximal and distal biliverdins, bind with stacked geometry in the active site. The nicotinamide ring of the NADP^+^ is located close to the reaction site on the proximal biliverdin, supporting that the hydride directly attacks this position of the proximal biliverdin. The results of mutagenesis studies suggest that a conserved Arg185 is essential for the catalysis. The distal biliverdin probably acts as a conduit to deliver the proton from Arg185 to the proximal biliverdin, thus yielding bilirubin.

## Abstract (web summary)

Biliverdin reductase (BVR) catalyses the last step in haem degradation. Here the authors present the crystal structure of cyanobacterial BVR bound to its substrate biliverdin and oxidised cofactor NADP^+^, which was used to propose the catalytic mechanism of this enzyme.

Biliverdin reductase (BVR, EC 1.3.1.24), first discovered in the 1960s (ref. 1), is the enzyme that converts biliverdin IXα (BV), a product of haem degradation, to bilirubin IXα (BR); this product is a bile pigment and a source of jaundice. Because BR forms intra-molecular hydrogen bonds, this reduction reaction causes the bilin pigment to change from hydrophilic to lipophilic^2^. Neonatal jaundice is a common disease in newborns; light therapy dramatically reduces the symptoms by changing the conformation of BR and breaking these intra-molecular hydrogen bonds, causing BR to become more hydrophilic and thus promoting its excretion^3^. Although over-accumulation of BR is toxic, at physiological concentrations BR is the major antioxidant responsible for protecting cells from H_2_O_2_ (ref. 4). Concomitant with H_2_O_2_-scavenging, BV produced by oxidation of BR is rapidly reduced back to BR by BVR, thereby amplifying its antioxidant efficiency 10,000-fold even though the BR concentration in tissues is quite low (∼20–50 nM: <0.1% level as compared with glutathione)^5^. The depletion of BVR by RNA interference markedly increases the level of reactive oxygen species and causes apoptotic cell death^4^. Thus, BVR plays a crucial role in the maintenance of intracellular redox balance.

BVR catalyses the reduction of the C10 double bond (γ-methene bridge) of BV using NAD(P)H to produce BR, a reaction in which the hydride (H^−^: a proton and two electrons) is donated by NAD(P)H (Fig. 1). This reaction also requires one additional proton to reduce the C10 double bond, but this proton donor has remained enigmatic for the past 50 years. In other words, the catalytic residue in the BVR protein moiety remains to be identified. Several crystallographic analyses have been performed to date: the structures of rat BVR in the apo-form and in complex with NAD^+^ have been reported by two independent groups^6^,^7^, and the structure of human BVR in complex with NADP^+^ has been deposited in the RCSB Protein Data Bank (PDB ID: 2H63). These crystallographic analyses revealed the binding site and mode of NAD(P)^+^. Although the substrate-binding site remained unclear, the residues located around the nicotinamide ring of NAD^+^ were considered strong candidates for the catalytic residues. Unexpectedly, however, extensive site-directed mutagenesis experiment revealed that alteration of these residues did not affect enzymatic activity. One exception was Tyr97 in rat BVR, which is located in the immediate vicinity of the nicotinamide ring; mutation of this residue reduces activity by 50% (ref. 7). Thus, Tyr97 promotes the enzymatic reaction, probably by indirectly influencing hydride transfer from NAD(P)H, but is not essential for catalysis.

Other characterizations of the mechanism underlying the BVR-catalysed reaction have been carried out, for example, investigations of substrate specificities using synthesized tetrapyrrole compounds^8^,^9^,^10^. These experiments examined a wide variety of tetrapyrrole compounds, including not only structurally modified alkyl side chains of tetrapyrrole but also BV isomers. The results revealed the invariant features of the BVR substrate: BVR exhibits a broad bilin substrate specificity, but strictly requires a propionate as the tetrapyrrole side chains^11^. Furthermore, a carboxyl group with a dissociable proton on the propionate side chain is essential, because its methyl- or azo-esterification completely abolishes BVR activity^12^.

Here we report the X-ray structures of the apo-, NADP^+^-bound and BV–NADP^+^ complex forms of BVR. This is the structure of the substrate–cofactor–enzyme ternary complex of BVR, revealing a binding mode in which two biliverdin molecules bind with stacked geometry in the active site. This structure and the results of site-direct mutagenesis can explain how BVR reduces BV to BR, thus resolving a longstanding mystery regarding the BVR reaction mechanism.

## Results

### Overall structure of apo-BVR from cyanobacteria

**Figure 2:**
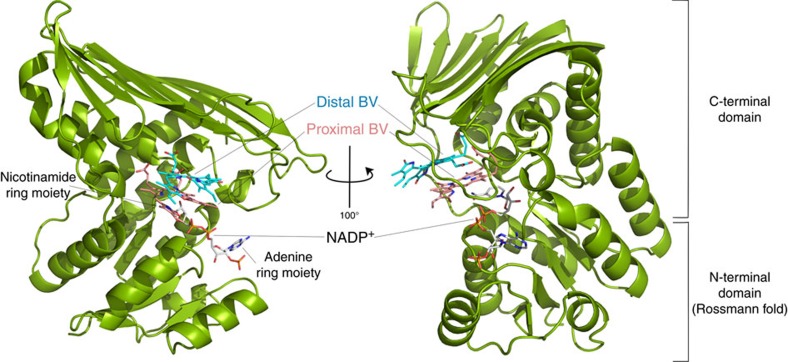
Overall structure of the *Syn* BVR complex. Ribbon representation of the crystal structure of *Syn* BVR in complex with BV and NADP^+^. The bound BVs and NADP^+^ are depicted with sticks. Right, View rotated by 100° about the vertical axis relative to left panel.

In this study, we used a cyanobacterial BVR from *Synechocystis* sp. PCC 6803 (*Syn* BVR)^13^ as a target for the structural analyses. The detailed properties of the purified *Syn* BVR, such as oligomeric state, are described in Supplementary Note 1 and Supplementary Fig. 1. All previous trials aimed at determining the structure of the substrate-binding site of rat BVR were unsuccessful^6^,^7^. *Syn* BVR is composed of 328 residues and shares more than 20% sequence identity with rat and human BVR proteins (Supplementary Fig. 2). We determined the crystal structure of *Syn* BVR by the single isomorphous replacement method coupled with anomalous scattering method using an Au-derivative crystal. The structure of apo-*Syn* BVR was refined at 2.1 Å resolution to *R* and *R*_free_ values of 0.200 and 0.239, respectively (Table 1). The asymmetric unit contains two BVR molecules. Although the electron density for apo-*Syn* BVR was mostly of high quality and continuous, the densities for the N-terminal region (residues 1–8) and C-terminal region (residues 326–328) were poorly defined. Therefore, these residues were not included in the model. *Syn* BVR consists of two structural domains (N-terminal and C-terminal domains), with the active sites in an obvious cleft between them (see Fig. 2). The N-terminal domain has a typical Rossmann fold, in which a parallel β-sheet (strands 1–5) is surrounded by five helices (helices 1–5). The C-terminal domain contains eight β-strands (strands 6–13) and six α-helices (helices 6–11). The overall topology of *Syn* BVR is nearly the same as those of the rat and human homologues, with two minor differences: *Syn* BVR has an additional short α-helix (α2) in the N-terminal domain, and has three additional β-strands in the C-terminal domain (Supplementary Fig. 2).

### NADP^+^ binding mode

**Figure 3:**
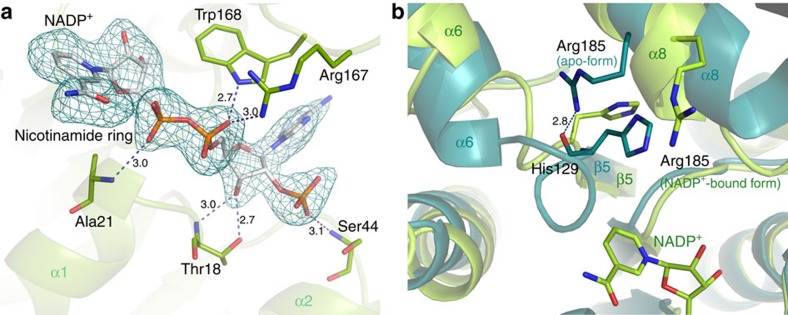
Electron density map around the bound NADP^+^ in the *Syn* BVR. (**a**) An omit *F*_o_–*F*_c_ difference map for NADP^+^ contoured at 2.0 σ (cyan) is overlaid on stick models of *Syn* BVR and the bound NADP^+^. Dashed lines indicate hydrogen bonds, and distances are represented in angstrom units. (**b**) Structural rearrangements in the active site upon NADP^+^ binding. The structures of the apo-form (teal green) and NADP^+^-bound form (light green) are superposed.

The *F*_o_–*F*_c_ electron density map calculated from the NADP^+^-soaked apo-form crystal clearly indicates that NADP^+^ binds in the cleft between the two domains (Fig. 3a). The density is visible for only one molecule in the asymmetric unit; thus, the two crystallographically independent BVR proteins in the asymmetric unit comprise one apo-form and one NADP^+^-bound form. The electron density of bound NADP^+^ was sufficiently clear that conformation and orientation could be explicitly determined. NADP^+^ is held by hydrogen bonds and hydrophobic interactions; Thr18, Ala21, Ser44, Arg167 and Trp168 form hydrogen bonding (Fig. 3a), whereas Trp41, Ile80, Glu101 and Tyr102 are involved in hydrophobic contact. Comparisons between the apo-form and the NADP^+^-bound form revealed small but significant structural rearrangements upon NADP^+^ binding: the N-terminal domain shifted ∼1.0 Å in order to expand the active site (Supplementary Fig. 3), and the loop between β5 and a part of helix α6 (residues 129–135) moved ∼2.0 Å away from the nicotinamide ring of the bound NADP^+^. Because of the steric hindrance configuration of the corresponding site in the apo-form would not able to bind NADP^+^; accordingly, these movements reflect an ‘induced fit' type of structural change. This induced fit was probably triggered by yet other structural rearrangements. Specifically, the regions around helix α8 differed from those in the apo-form (Fig. 3b): the starting point of helix α8, located near helix α6 as well as the nicotinamide ring of the bound NADP^+^ (but not directly interacting with NADP^+^), was altered. In the apo-form, the guanidine group of Arg185 in helix α8 formed a hydrogen bond with the carbonyl oxygen of His129 in the loop. In the NADP^+^-bound form, the hydrogen bond between Arg185 and His129 was broken, and the side chain of the Arg185 rotated toward the active site due to the overall rearrangement. These structural changes confer not only substrate-binding ability, but also catalytic activity, on BVR (see below).

### Substrate BV binding mode

**Figure 4:**
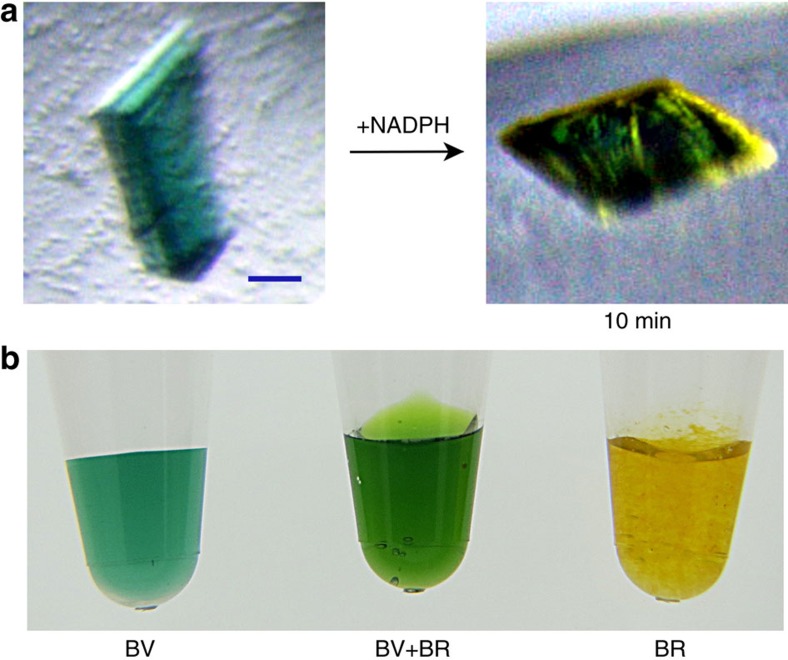
The enzymatic reaction in the crystalline state. (**a**) The BV reduction could proceed in the crystal by adding NADPH. Right, 10 min after the reaction. The bluish-green colour in the crystal (left) is derived from the bound BV. The yellow–green colour in the crystal (right) is probably derived from the mixture of BV and BR. Scale bar, 20 μm (**b**) The colours of the pigments in solution, provided as a reference: BV (left); mixture of BV and BR (middle) and BR (right). Final concentrations of all pigments were adjusted to 0.5 mM.

**Figure 5:**
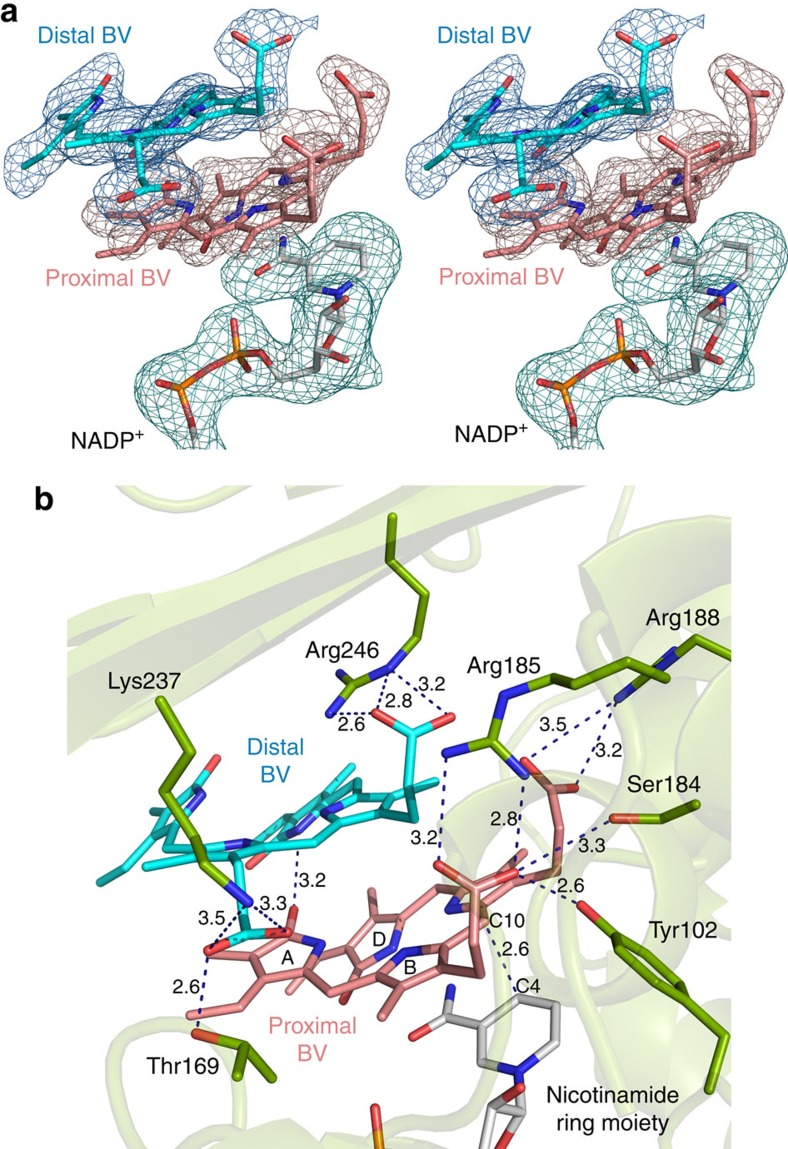
The binding modes of the BVs and NADP^+^ in *Syn* BVR. (**a**) Stereo view of the electron density map around the bound two BVs and NADP^+^ in the *Syn* BVR. An omit *F*_o_–*F*_c_ difference map for BVs (blue and salmon pink) and NADP^+^ (cyan) contoured at 2.0 σ is overlaid on the stick models of the bound BVs and the bound NADP^+^. The view direction is same as in right panel in Fig. 2. (**b**) The environment around the bound two BVs in the *Syn* BVR. The bound BVs, NADP^+^ and the residues involved in the BV binding are shown with the stick. Dashed lines indicate hydrogen bonds.

We successfully obtained BV-bound BVR crystals by the co-crystallization screening, and the resulting crystals were a bluish-green colour (Fig. 4a). The BV-bound crystals belong to a different space group (*P*2_1_) than those of the apo- and NADP^+^ soaked forms (*P*2_1_2_1_2_1_), and the asymmetric unit contains four BVR molecules (A to D molecules). Surprisingly, two BV molecules are bound to the catalytic cleft between the two domains (Fig. 2), and adopt a stacked configuration along with the tetrapyrrole plane (Fig. 5). In each of the four BVR molecules, electron densities derived from the tetrapyrrole moiety of two BV molecules are clear (Fig. 5a). NADP^+^ molecules were also bound by all four BVR molecules, although we did not add the NADP^+^ reagent during the purification and crystallization procedures. Therefore, the NADP^+^ in the BV-bound crystal was derived from *E. coli*, and their binding configurations are thought to represent the authentic form. The configuration of NADP^+^ in the ternary complex was identical to that in the NADP^+^-soaking crystal. This structure is the substrate–cofactor–enzyme ternary complex reported for BVR. One BV (the proximal BV) was bound in close vicinity of the nicotinamide ring, and the other one (the distal BV) was located on the upper stack with the proximal BV (Fig. 5).

The superimposition of four BVR molecules in the asymmetric unit showed that the binding configurations of NADP^+^ and proximal and distal BVs are almost the same. The proximal BV interacts with the distal BV by hydrophobic and van der Waals interactions. The propionate groups form two salt bridges with amino acid residues (Fig. 5b), one interacting with Arg185, and the other interacting with Arg188. These interactions between the propionate group and the surrounding residues fix the propionate side chains to these positions. The C10 double bond in the proximal BV, which is to be reduced, faces the nicotinamide C4 carbon (origin of the hydride) of the NADP^+^; the distance between the C10 carbon of the proximal BV and the C4 carbon of the NADP^+^ is 2.6 Å, and the angle between the C10 and nicotinamide ring was ∼120°. This configuration of the proximal BV appears suitable to accept the hydride from the nicotinamide ring, and strongly suggests that the hydride is directly transferred to the C10 carbon in the proximal BV.

In the distal BV, the binding configuration could be also defined based on the electron density map (Fig. 5a). The configurations of all four distal BVs in the asymmetric unit are almost identical. The tetrapyrrole plane of the distal BV is located nearly parallel to that of the proximal BV, and the directions of the propionate side chains of each BV are the same. The distal BV is located ∼4 Å above, translated by ∼2.5 Å to the solvent side and rotated ∼45° relative to the proximal BV (Fig. 5b). Consequently, the B-ring in the distal BV is located just above the A-ring of the proximal BV. One propionate side chain forms a salt-bridge with the Arg246, and the other side chain forms a hydrogen bond with Thr169. In addition, the pyrrole nitrogen of the D-ring in the distal BV might form a hydrogen bond with the lactam group (C=O) of the A-ring in the proximal BV; the distance between them was ∼3.2 Å.

### Steady-state kinetics of *Syn* BVR

We next investigated the catalytic residue based on this structure. One proton and two electrons are served from the NAD(P)H directly to the C10 double bond in the proximal BV; thus, the other unidentified catalytic residue must be located close to the proximal BV. The structure, however, revealed that only the hydroxyl group of Tyr102 interacts with C10, and this interaction is weak (∼3.5 Å, either via van der Waals force or CH/O hydrogen bond). Previous mutagenesis studies of *Syn* and rat BVRs clearly indicated that the tyrosine residue (Tyr97 in rat BVR) is not a direct proton donor^6^,^7^,^14^. Although the H84A and D287A mutations in *Syn* BVR dramatically reduced its activity^14^, these residues have no direct interaction with bound NADP^+^ or bound BV (Supplementary Fig. 4). Accordingly, we excluded His84 and Asp287 as candidates for the proton donor (see details in Supplementary Note 2 and Supplementary Fig. 4). Therefore, we further investigated the possible candidates for the catalytic residues surrounding the proximal and distal BVs. Six residues were considered as possible proton donors (within ∼3.5 Å): the hydroxy groups of Thr169 and Ser184, and the amino groups of Arg185, Arg188, Lys237 and Arg246 (Fig. 5b).

Steady-state kinetics, monitored using the *Syn* BVR proteins containing mutations in these candidate proton donor residues, revealed the plausible catalytic residues (Table 2). The enzymatic activity of the mutation at Tyr102 was reduced to ∼40% in terms of *k*_cat_ value. This behaviour was in agreement with that of rat BVR (Tyr97 mutant)^7^. Notably, the turnover rates were dramatically decreased relative to that of the wild-type protein by introduction of mutations at Arg185 (R185A and R185K): *k*_cat_/*K*_m_ was reduced to ∼0.25%, whereas their affinity for the substrate (*K*_m_) was not significantly altered. These results also indicated that the guanidine group of the arginine residue in this position was invariant for the enzymatic reaction, as it was not able to substitute for positively charged amino group of the lysine residue. This arginine residue is completely conserved among BVR homologues, including rat (Arg171) and human BVR (Arg172) (Supplementary Fig. 2).

Next, we examined the enzymatic activity of rat and human BVRs by replacing the arginine at this site with other residues. As a result, in both rat and human BVRs, the *k*_cat_/*K*_m_ values were remarkably decreased. In human BVR, the R172E and R172K mutations completely abolished the enzymatic activity. In the cofactor-bound state, although the position of the guanidine group of this arginine residue was almost identical among *Syn*, human, and rat BVRs (Supplementary Fig. 5), the conformation of its side chains had little variation (for example, torsion angles of Cβ and Cγ atoms). Taken together, these data indicate that this arginine residue is the common catalytic residue of BVRs.

### The BV binding to *Syn* BVR in solution states

**Figure 6:**
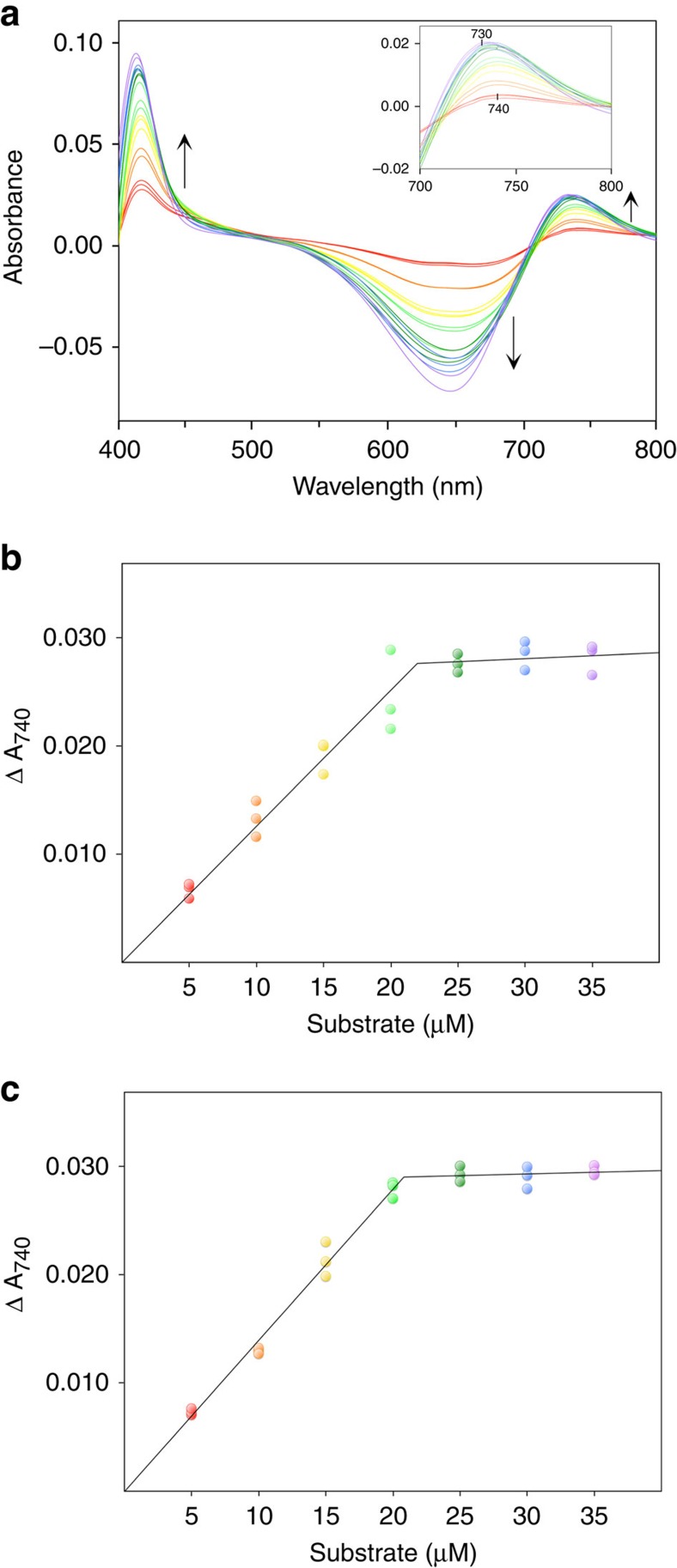
Determination of the stoichiometry of BV binding to *Syn* BVR. (**a**) BV titration of *Syn* BVR (10 μM), as determined from the difference absorption spectra acquired in the presence of NADP^+^. Arrows indicate trends of spectral changes. (**b**) Titration curve of *Syn* BVR observed at 740 nm. (**c**) Titration curve of the *Syn* BVR R185A mutant. Measurements were performed in triplicate.

To obtain further insight into the substrate-binding stoichiometry in solution, we measured BV binding to *Syn* BVR by optical titration. The BV absorption spectrum was slightly altered upon BVR binding in the presence of NADP^+^. Difference spectra (that is, in *Syn* BVR present minus *Syn* BVR absent) exhibited a negative peak at 650 nm and positive peaks at 420 and 740 nm (Fig. 6a). BV titration revealed that all of the difference peaks blue-shifted by ∼10 nm. Peak amplitudes increased with BV concentration up to a twofold excess of BVR. The titration curve at 740 nm clearly indicated that two BV molecules bound to *Syn* BVR (Fig. 6b). This BV titration result agreed closely with the crystal structure. We also performed BV titration using the *Syn* BVR R185A mutant protein. The resultant titration curve confirmed that two BV molecules bound to *Syn* BVR R185A (Fig. 6c). Thus, the arginine residue in this position is not necessary for the two BV stacked binding, but is essential for the catalytic function.

To determine whether two-substrate binding is common among mammalian BVRs, we performed a titration in human and rat BVRs (Supplementary Fig. 6). The titration curve indicated that two BV molecules bound in both human and rat BVRs. Therefore, the mode of substrate binding is common among all BVRs.

## Discussion

In this study, we experimentally determined the substrate-binding configurations in BVR concomitant with that of NADP^+^. To the best of our knowledge, parallel stacking of two tetrapyrrole molecules is quite rare binding configuration. Many multi-tetrapyrrole proteins are present, including photosynthesis complexes^15^,^16^ and cytochromes^17^. However, even if the tetrapyrrole compounds are located close to each other in these proteins, the two tetrapyrrole rings are not stacked, and the propionate side chain moieties are crossed or stacked in most cases. By contrast, in the BVR structure revealed in this study, the tetrapyrrole rings in the proximal and distal BVs are nearly parallel. Furthermore, the tetrapyrrole rings are located close to each other (within ∼4 Å).

Given that the enzymatic activity of BVRs is inhibited by excess BV^18^, this unprecedented configuration raises the possibility that this structure reflects a substrate-inhibited state. However, in the procedure of the co-crystallization in this study, we never added a molar excess of substrate, and the crystallization drops contained equimolar amounts of BVR and BV. Indeed, the enzymatic reaction of BV reduction could be induced to occur, even in the crystalline state, by addition of NADPH (see Fig. 4a). The colour of the crystal 10 min after the reaction was quite similar to that of a solution mixture of BV and BR (see Figs 4a,b), even though the crystal gradually melted. Moreover, we confirmed that BV could be rapidly (as in the mixing time) reduced to BR by addition of NADPH in a solution identical to the one used for crystallization. Thus, the structure presented here represents the active state.

The mutagenesis and kinetics experiments revealed that an arginine residue that is completely conserved among BVRs is probably the proton donor residue (see Table 2 and Supplementary Fig. 1). Intriguingly, Arg185 is located far from the C10 double bond of the reducing site of the proximal BV (>5.7 Å) and the distal BV (>6.0 Å) (see Fig. 5b). That is, the proton is not able to transfer directly to the reducing site. Instead, the guanidine group of Arg185 formed hydrogen bonds with the carboxy group of the propionate side chain of the proximal BV (within 2.8 Å in all molecules of the asymmetric unit); thus, the Arg185 must first donate its proton to the propionate side chain of the proximal BV.

Because of their chi-angle rotation restraints, it would be extremely difficult for the propionate side chains of the proximal BV to transfer the proton to reduced site. The parallel stacked configuration of the proximal and distal BVs indicates that the proton from Arg185 in the propionate side chain of the proximal BV can easily transfer to the propionate side chain in the distal BV. Although the propionate side chain of the distal BV is also located close to Arg185 (∼3.1 Å), and probably the proton could be directly transferred from Arg185, a hydrogen bond may not be formed due to the parallel configuration between the amino acid residue and the side chain. The propionate side chain in the distal BV is in a suitable location for transferring the proton to the proximal BV: it can approach within 2.6 Å of the C10 carbon, as well as the pyrrole nitrogen in the B-ring in the proximal BV (<2.5 Å), via rotation of the propionate side chain of the distal BV.

**Figure 7:**
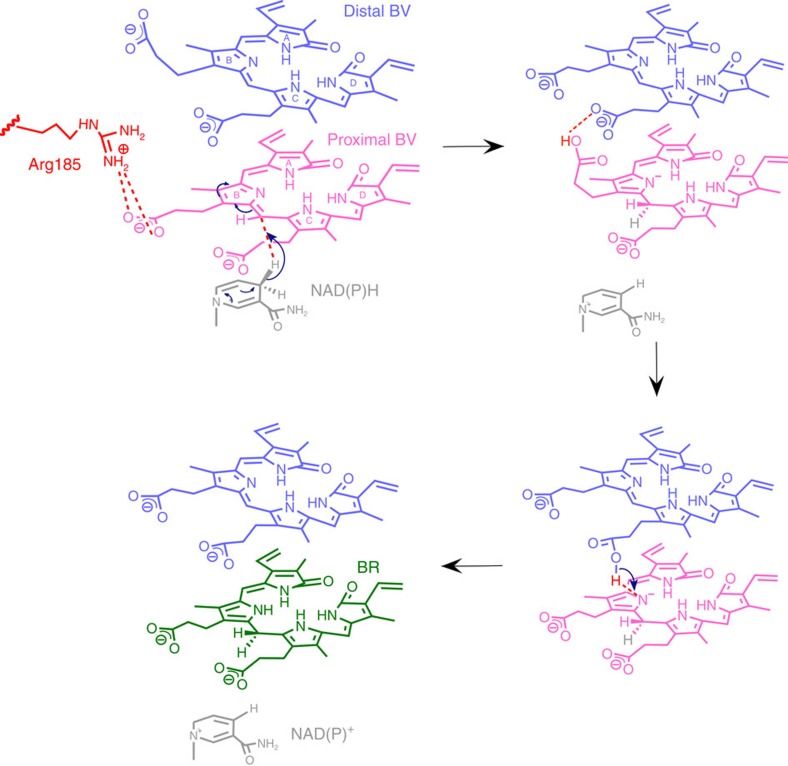
Proposed mechanism of the BVR reaction. A hydride from NAD(P)H is directly transferred to the proximal BV, initiating the reduction of the proximal BV. The propionate side chain of the proximal BV accepts a proton from Arg185, subsequently, the proton is transferred to the propionate side chain in the distal BV, and the protonated propionic acid group in the distal BV moves toward the reducing site in the proximal BV to transfer the proton. Finally, the proximal BV accepts the proton to produce BR. Arrows in the nicotinamide and pyrrole rings indicate the electron flow, and in the other positions indicate the proton or hydride transfer.

Taken together, these observations suggest a plausible proton pathway for conversion of BV to BR (Fig. 7): a hydride from NAD(P)H is directly transferred to the C10 double bond of the proximal BV, initiating the reduction of the proximal BV. The propionate side chain of the proximal BV accepts a proton from Arg185, thereby abolishing the electrostatic interaction between Arg185 and the propionate and increasing the flexibility of the side chain. Subsequently, the proton is transferred to the propionate side chain in the distal BV, and the protonated propionic acid group in the distal BV moves toward the reducing site in the proximal BV to transfer the proton. Although the exact site to be transferred to the proton could not be determined by the present structure, the B-ring in the proximal BV is the most reasonable site since the propionate side chain in the distal BV can be located close to the B-ring in the proximal BV. Finally, the proximal BV accepts the proton to produce BR.

The reaction mechanism proposed here could explain not only why BVR strictly requires the propionate as tetrapyrrole side chain^11^, but also why a carboxyl group with a dissociable proton on the propionate side chain is essential^12^. Mantle's group performed sophisticated kinetic analysis of BVR, and found that *Syn* BVR obeys an ordered steady-state kinetic mechanism, with NADPH the first to bind and NADP^+^ the last to dissociate^14^. Based on this concept, we proposed a reaction mechanism for BVR. Following NADPH binding, the reaction is likely to start with hydride transfer from NAD(P)H, because in time-course ultraviolet–visible spectra we have never observed a peak derived from BVH^+^, as seen in other BV-related enzymes^19^,^20^. The propionate side chain in another tetrapyrrole compound, protochlorophyllide, serves as proton donor for its self-reduction at the C17=C18 double bond to form chlorophyllide *a*^21^. Thus, it is feasible that the proton donation function of the propionate side chain of BV is involved in the reduction reaction. QM/MM calculations for human BVR (BVR-A) and a related enzyme (BVR-B) have shown that the water molecule might be a proton donor^22^,^23^, although the working hypothesis is that BVR binds one biliverdin molecule. We hope that by assuming binding of two biliverdin molecules, the QM/MM calculations can be used to reveal the detailed reaction mechanisms of BVR.

A structural and mechanistic understanding of the BVR reaction may enable the development of new inhibitors that target severe jaundice. In particular, the active pocket, where two BVs bind with the unique stacked geometry, is a promising target for design of drugs with minimal risk of deleterious side effects.

## Methods

### Purification and crystallization of *Syn* BVR

The plasmid pET15b-*Syn bvr*^24^ was used to overproduce N-terminal hexa-histidine tagged *Syn* BVR in the C41(DE3) strain (Lucigen) of *E. coli*. The cells were grown in Terrific-broth (TB) medium containing ampicillin (50 μg ml^−1^) at 28 °C, and overproduction was sustained overnight under shaking at 28 °C after induction with 1 mM isopropyl β-D-thiogalactopyranoside (IPTG). The N-terminal His-tagged *Syn* BVR was purified using nickel affinity gel (Nacalai Tesque) according to the manufacturer's protocol. The non-tagged *Syn* BVR was prepared by digestion of the N-terminal His-tagged *Syn* BVR with 10 units of thrombin (GE Healthcare) per milligram of the protein in PBS buffer (10 mM Na_2_HPO_4_, 1.8 mM KH_2_PO_4_, 2.7 mM KCl and 140 mM NaCl) at 20 °C for 48 h. Non-tagged *Syn* BVR was separated from remaining His-tagged *Syn* BVR by mixing it again with the nickel affinity gel. The flow-through fraction containing the non-tagged *Syn* BVR was concentrated with a VIVASPIN filter (GE Healthcare), and applied onto a HiPrep 16/60 Sephacryl S-200 HR gel filtration column (GE Healthcare) equilibrated in PBS buffer. The elution profile was monitored at absorption at 280 nm, and its purity was confirmed by SDS–PAGE.

Purified non-tagged *Syn* BVR in PBS buffer was crystallized by the hanging-drop vapour diffusion method. In the initial crystallization screening, drops consisting of 1 μl protein solution mixed with 1 μl precipitant solution. Final optimized crystallization conditions were as follows: 12 mg ml^−1^ protein in 50 mM Tris-HCl (pH 7.8) containing 150 mM NaCl and the reservoir solution was composed of 15% PEG 4,000, 50 mM Tris-HCl (pH 7.25), 0.2 M sodium acetate and 0.2 mM Cymal-2 (Hampton Research).

### Data collection and structure determination of apo-*Syn* BVR

The crystals were cryoprotected with a reservoir solution containing 12.5% glycerol for a few seconds, and then flash-cooled in a cryostream. The complete apo-*Syn* BVR data set was collected on an ADSC Quantum 4R detector with synchrotron radiation at a wavelength of 1.000 Å at BL38B1 beamline of SPring-8 (Hyogo, Japan). Intensity data were processed and scaled using the HKL2000 software package^25^.

Gold derivatives were obtained by soaking apo-*Syn* BVR crystals for 2 h in reservoir solution containing 10 mM potassium dicyanoaurate (I). The Au-soaked crystal was cryoprotected with reservoir solution containing 10% glycerol and flash-frozen in liquid nitrogen. X-ray diffraction data were collected at BL32XU beamline of SPring-8 and processed using HKL2000. Experimental phases were obtained from the Au-soaked crystal by the single isomorphous replacement method coupled with anomalous scattering (SIRAS) using AutoSol in PHENIX^26^. The model was built manually in COOT^27^, and refined in PHENIX. Secondary structures were assigned using PROMOTIF^28^, and the geometry of the final model was analysed using MolProbity^29^. Data collection and refinement statistics are summarized in Table 1.

### Structure determination of NADP^+^-bound *Syn* BVR

Cofactor-bound crystals were prepared by soaking crystals for 20 h in a reservoir solution containing 10 mM NADP^+^. The NADP^+^-soaked crystal was transferred to a cryo-protectant solution containing 10% glycerol and flash-frozen in liquid nitrogen. The data sets were collected at BL44XU beamline of SPring-8 and processed with the XDS package^30^.

Because the NADP^+^-soaked *Syn* BVR crystals were nearly isomorphous with the apo-form crystal, the structure of NADP^+^-bound *Syn* BVR was determined using the structure of apo-*Syn* BVR (PDB ID: 5B3T). Refinement was done using PHENIX^26^ and the model was revised manually in COOT^27^. In this step, the NADP^+^ was clearly visible in the electron density map. After one cycle of water picking, energy minimization refinement, and temperature-factor refinements, the model of the NADP^+^ was fitted to the *F*_o_–*F*_c_ difference map in the active pocket. Finally, energy minimization and temperature-factor refinements in PHENIX were applied to the model including NADP^+^. Refinement statistics are summarized in Table 1.

### Structure determination of *Syn* BVR in complex with BV and NADP^+^

*Syn* BVR in complex with BV and NADP^+^ was crystallized using the sitting-drop vapour-diffusion method. Crystallization drops made using Mosquito (TTP Labtech) consisting of 0.1 μl protein solution and 0.1 μl reservoir solution were equilibrated against 50 μl reservoir solution. Initial crystallization trials were done with commercially available sparse-matrix screening kits. The bluish-green small crystals grew at 20 °C. Crystallization conditions were as follows: 20 mg m1^−1^ (0.52 mM) protein in PBS buffer containing 0.52 mM BV; reservoir solution consisting of 19% PEG 4000, 30 mM Tris-HCl (pH 8.2), 50 mM MgCl_2_ and 10 mM BaCl_2_. Crystals grew to maximum dimensions of 0.08 × 0.05 × 0.01 mm.

The crystal was cryoprotected with the reservoir solution containing 20% glycerol and flash-frozen in liquid nitrogen. X-ray diffraction data were collected on a Rayonics MX300HE detector at BL44XU beamline of SPring-8 and processed with HKL2000. The crystal belongs to the monoclinic space group *P*2_1_, and a complete data set was collected to a resolution of 2.6 Å (Table 1). The structure was solved by the molecular replacement method using the model of apo-*Syn* BVR (PDB ID: 5B3T) as the search probe, excluding solvent molecules. Rotational and translational searches with MOLREP^31^ in the CCP4 package^32^ using the data in the resolution range 15.0–4.0 Å located four molecules in the asymmetric unit. The coordinates of the model and the temperature factors were refined using PHENIX^26^ and manually revised with COOT^27^. After several cycles of refinement and model adjustment, BV, NADP^+^ and water molecules were included in the model. The coordinates, topologies, and parameters of BV were generated using the PRODRG2 server^33^. Diffraction data and refinement statistics are summarized in Table 1.

### Expression and purification of rat BVR

The plasmid pET21a-rat *bvr*^34^ was used to overproduce C-terminal His-tagged rat BVR in the BL21(DE3) strain (Novagen) of *E. coli*. The cells were grown overnight in TB medium containing ampicillin (50 μg ml^−1^) at 28 °C without IPTG induction. The cells were collected by centrifugation, and the cell pellets were frozen at −80 °C.

The C-terminal His-tagged rat BVR was purified using nickel affinity gel (Nacalai Tesque). After His-tagged rat BVR was eluted from the gel, the sample solution was exchanged to the PBS buffer by dialysis.

### Expression and purification of human BVR

The human BVR gene was constructed from synthetic DNA (Eurofins Genomics) with codon optimization for *E. coli*. The human BVR gene was cloned into pET21a(+) (Novagen) in *E. coli* strain BL21(DE3) (Novagen) to produce C-terminal His-tagged human BVR. His-tagged human BVR was expressed and purified as described for His-tagged rat BVR.

### Site-directed mutagenesis

Site-directed mutagenesis was carried out using KOD-plus-Mutagenesis Kit (Toyobo), pET15b containing *Syn* BVR gene or pET21a(+) containing human or rat BVR gene as a template, and the primers listed in Supplementary Table 1. Sequence analysis verified that the resultant constructs were free of errors.

### Enzymatic assay

*Syn* BVR activity was measured as previously reported^13^ by monitoring the increase in absorbance at 450 nm (due to appearance of BR) using a V630BIO spectrometer (JASCO). Briefly, the reaction mixture contained 10 μg purified BVR (0.27 μM), 100 mM citrate buffer (pH 5.75), 100 μM NADPH and various concentration of BV. The total volume was 1 ml, and the reaction was initiated by addition of NADPH. The Michaelis constant values for BV were determined using reaction mixtures containing various concentrations of BV, and initial velocity data were analysed by non-linear curve fitting using GraphPad Prism 6 (GraphPad Software). The human and rat BVR assays used the same conditions as for *Syn* BVR, except that the buffer contained 100 mM Tris-HCl (pH 8.75).

### Determination of protein and BV concentrations

Protein concentrations were determined by UV absorption using a molar extinction coefficient (*ɛ*_280_ values of 41,370 M^−1^ cm^−1^ for the *Syn* BVR, 25,440 M^−1^ cm^−1^ for the human BVR and 23,950 M^−1^ cm^−1^ the for rat BVR, respectively) calculated from the amino acid sequence using the ExPASy ProtParam server^35^. BV was dissolved in a small amount of 0.1N NaOH and diluted into 100 mM Tris-HCl (pH 7.8); the concentration of BV was determined in HCl(5%)/methanol using an *ɛ*_696_ value of 30,800 M^−1^ cm^−1^ (ref. 13).

### BV titration assay

BV binding stoichiometry was estimated by optical titrations in PBS buffer containing 5 mM NADP^+^ at 28 °C. BV was added in 5 μM increments to both the sample and the reference cuvettes; the former contained 10 μM *Syn* BVR, whereas the latter contained no *Syn* BVR. Optical absorption spectra ranging from 400 to 800 nm were recorded at least 10 min after each addition of BV using a V630BIO spectrometer. Titration assays for human and rat BVR used the same conditions as for *Syn* BVR, except that the buffer contained 200 mM Tris-HCl (pH 8.75).

### Size-exclusion chromatography

Size-exclusion chromatography was conducted using Sephacryl 200 HR 16/60 (GE Healthcare) columns equilibrated with PBS buffer (140 mM NaCl, 2.5 mM KCl, 10 mM Na_2_HPO_4_, 1.8 mM KH_2_PO_4_) at 20 °C. The calibration proteins [β-amylase (200 kDa), alcohol dehydrogenase (150 kDa), BSA (66 kDa), carbonic anhydrase (29 kDa) and cytochrome *c* (12.4 kDa)] were applied to the column.

### Dynamic light scattering

Dynamic light scattering analysis was performed using a DynaPro99 Dynamic Light Scattering system (Protein Solutions). The wild-type *Syn* BVR (1 mg ml^−1^) was centrifuged at 20,400*g* for 30 min to remove large aggregates prior to the experiments. Twenty readouts were averaged to obtain the mean and s.d. of sample molecular weights.

### Enzymatic assay for NADH-dependent BVR activity

NADH-dependent BVR activity was measured by monitoring the increase in absorbance at 450 nm. The reaction mixture contained 10 μg purified BVR (0.27 μM), 100 mM citrate buffer (pH 5.75), 700 μM NADH, 20 μM BV and various concentration of sodium phosphate. Initial velocity data were analysed by non-linear curve fitting using GraphPad Prism 6 (GraphPad Software). In this measurement, great care was taken to control pH, because the activity is highly sensitive to pH.

### Thermal-shift assays of *Syn* BVR

Thermal-shift assays were performed on a CFX96 Real-Time PCR Cycler (Bio-Rad Laboratories). In these experiments, 1 μl of SYPRO Orange (Sigma-Aldrich, diluted from 5,000 × stock into Milli-Q), 8 μl protein (1.0 mg ml^−1^), and 1 μl buffer (1 M citrate buffer (pH 5.75)) were mixed on ice in a white 96-well PCR plate (Bio-Rad Laboratories). To evaluate the effect of NADP^+^ binding, 1 μl of 5,000 × SYPRO Orange, 8 μl protein (1.0 mg ml^−1^) and 1 μl buffer (1 M citrate buffer (pH 5.8)) containing 10 mM NADP^+^ were mixed. Fluorescence was measured as temperature increased from 25 to 85 °C in 0.5 °C steps (excitation, 450–490 nm; detection, 560–580 nm). All measurements were performed three times. Data evaluation and melting-point determination were conducted using the Bio-Rad CFX Manager software.

### Data availability

Coordinates and structure factors for apo-, NADP^+^-bound and BV–NADP^+^ complex forms of BVR have been deposited in the Protein Data Bank under accession codes 5B3T, 5B3U and 5B3V, respectively. All additional experimental data are available from the corresponding author on reasonable request. The PDB accession codes 1LC3 and 2H63, UniProt accession codes P53004, P46844, P72782, Q8YPP4 and Q9CY64 were used in this study.

## Additional information

**How to cite this article:** Takao, H. *et al*. A substrate-bound structure of cyanobacterial biliverdin reductase identifies stacked substrates as critical for activity. *Nat. Commun.* **8,** 14397 doi: 10.1038/ncomms14397 (2017).

**Publisher's note**: Springer Nature remains neutral with regard to jurisdictional claims in published maps and institutional affiliations.

## Supplementary Material

Supplementary Information Supplementary figures, supplementary table, supplementary note, supplementary methods and supplementary references.

Peer Review Article

## Figures and Tables

**Figure 1:**
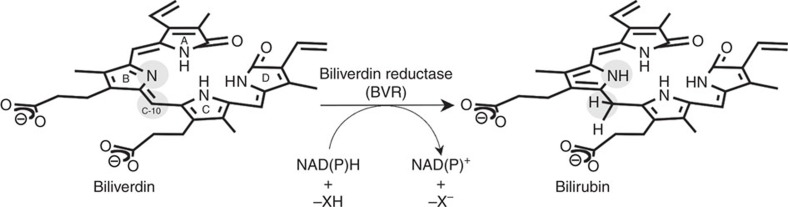
Reaction catalysed by BVR. The C10 double bond (γ-methene bridge) in BV is reduced by hydride from the cofactor, and the pyrrole nitrogen of the ring is protonated (hydride and proton are depicted on grey circular backgrounds on the product, BR).

**Table 1:** Data collection and refinement statistics.

	**Apo-form**	**Au derivative**	**NADP**^**+**^ **complex**	**Biliverdin-NADP**^**+**^ **complex**
Data collection*				
Space group	*P*2_1_2_1_2_1_	*P*2_1_2_1_2_1_	*P*2_1_2_1_2_1_	*P*2_1_
Cell dimensions				
* a, b, c* (Å)	58.2, 88.5, 133.6	59.0, 88.5, 134.4	59.1, 85.6, 135.0	55.5, 100.4, 138.0
* α, β, γ* (°)	90.0, 90.0, 90.0	90.0, 90.0, 90.0	90.0, 90.0, 90.0	90.0, 95.5, 90.0
* *Resolution (Å)	43.90–2.10 (2.23–2.10)	48.77–2.10 (2.20–2.10)	48.62–2.70 (2.86–2.70)	49.62–2.60 (2.69–2.60)
* *No. unique reflections	40,823	78,417	19,500	46,736
* R*_merge_	0.097 (0.429)	0.108 (0.492)	0.123 (0.664)	0.094 (0.458)
* I*/*σI*	18.2 (4.0)	7.4 (3.7)	15.3 (3.5)	7.4 (3.2)
* *Completeness (%)	99.0 (95.1)	97.2 (84.5)	99.8 (99.0)	99.9 (100.0)
* *Redundancy	6.9(3.6)	5.8 (3.4)	10.0 (10.1)	4.2 (4.2)
				
*Refinement*				
* *Resolution (Å)	43.90–2.10 (2.23–2.10)		48.62–2.70 (2.86–2.70)	49.62–2.60 (2.69–2.60)
* R*_work_/*R*_free_†	0.200/0.239		0.223/0.271	0.208/0.258
* *No. atoms	5,320		5,092	10,756
* *Protein	5,020		4,974	9,993
* *Ligand NADP^+^	—		48	192
* *Ligand BV	—		—	344
* *Ligand PO_4_	20		20	—
* *Water	280		50	227
* *Average *B* factors (Å^2^)	23.9		49.0	27.8
* *Protein	23.8		49.1	27.5
* *Ligand NADP^+^	—		43.4	26.5
* *Ligand BV	—		—	39.4
* *Ligand PO_4_	31.8		57.2	—
* *Water	24.3		43.7	24.3
* *r.m.s.d.'s				
* *Bond lengths (Å)	0.002		0.003	0.005
* *Bond angles (°)	0.641		0.721	1.761
* *Ramachandran plot				
* *Favoured (%)	98.9		97.0	97.4
* *Allowed (%)	1.1		3.0	2.6
* *Outliers (%)	0		0	0

^*^Values in parentheses correspond to the highest-resolution shell.

^†^*R*_free_ is the *R*-factor calculated for 5% of the data set not included in refinements.

**Table 2:** Kinetic parameters of wild-type and mutant proteins of *Syn*, human and rat BVRs* †.

**Variant**	***k***_**cat**_ **(s**^**−1**^**)**	***K***_**m**_ **(μM)**	***k***_**cat**_**/*****K***_**m**_ **(s**^**−1**^ **μM**^**−1**^**)**
*Synechocystis* sp. PCC 6803			
Wild-type	7.1±0.2	1.8±0.3	3.9
Y102F	2.7±0.07	4.0±0.3	0.7
T169A	13.1±0.5	3.4±0.5	3.9
S184A	11.3±0.5	1.9±0.4	5.9
R185A	0.7±0.02	6.9±0.6	0.1
R185K	0.9±0.05	12.6±1.5	0.07
R188A	13.4±0.4	3.3±0.4	4.1
K237A	23.7±2.1	4.8±1.3	4.9
R246A	11.0±0.6	5.1±0.8	2.2
			
*Human*			
Wild-type	94.8±31.4	20.7±10.1	4.6
Y98F	16.8±0.5	3.8±0.3	4.4
R172A	17.9±6.3	87.7±35.7	0.2
R172E	N.D.	N.D.	—
R172K	N.D.	N.D.	—
			
*Rat*			
Wild-type	69.3±8.5	4.6±1.1	15.1
Y97F	13.6±0.6	11.7±1.0	1.2
R171A	6.0±0.2	24.4±1.5	0.2
R171E	0.7±0.2	45.7±15.4	0.02
R171K	14.7±8.5	163.3±103.2	0.1

^*^Errors (s.d.) are calculated from triplicate measurements.

^†^N.D., not detectable.
