# Supplementary material for: A substrate-bound structure of cyanobacterial biliverdin reductase identifies stacked substrates as critical for activity
Source: Nat Commun. 2017 Feb 7;8:14397. doi: 10.1038/ncomms14397 (PMC5309722; doi:10.1038/ncomms14397)
Supplement: Supplementary Information — Supplementary figures, supplementary table, supplementary note, supplementary methods and supplementary references. [file ncomms14397-s1.pdf]

Supplementary Note 1. Enzymatic properties of the purified *Syn* BVR

The expression vector pET15b-*Syn bvr* allowed us to routinely prepare 15 mg of electrophoretically homogenous *Syn* BVR from 2.5 L of TB-medium cultures of *E. coli* C41(DE3) cells. When prepared by the thrombin treatment approach, the enzyme has three additional residues at the N-terminus (Ser-Gly-His) but lacks the His-tag. SDS-PAGE analysis of purified *Syn* BVR revealed a major Coomassie blue–stained band at ~36 kDa (Supplementary Fig. 1(a)), consistent with the mass of *Syn* BVR estimated from its DNA sequence. The purity of *Syn* BVR was determined to be greater than 95% using Coomassie blue–stained gels. The molecular size of the *Syn* BVR was estimated to be ~36 kDa by gel-filtration chromatography (Supplementary Fig. 1(b)). Dynamic light scattering analysis estimated the size to be 35.4 kDa with monomodal dispersity (polydispersity = 8%). In addition, the crystal structures in this study revealed that all structures, including the apo-, $NADP^+$-bound, and BV-complex of *Syn* BVR, were in the monomeric state. Therefore, we concluded that purified *Syn* BVR existed as a monomer under our experimental conditions.

It has been reported previously that *Syn* BVR expressed in *E. coli* apparently formed dimers[1]. There is a discrepancy between our purified *Syn* BVR and previously reported *Syn* BVR in the oligomeric state. However, we know a great deal about protein with two oligomeric states. For example, cytochrome *c*, a heme electron carrier protein, was long believed to be a monomeric protein. Recently, however, new findings demonstrated that the monomeric state of cytochrome *c* could be altered to a dimeric form by treatment with ethanol or refolding with guanidinium *in vitro*[2]. Furthermore, its dimeric states could be also detected in the cell[3]. In regard to *Syn* BVR, these results raise the possibility that both the monomeric and dimeric states might reflect unknown physiological functions of *Syn* BVR. Consistent with this, recent work revealed additional functions of human BVR, including kinase and transcription factor (DNA binding) activities[4,5].

Phosphate ions enhance the NADH-dependent activity of human BVR[6]. We observed similar enhancement of *Syn* BVR activity by inorganic phosphate (Supplementary Fig. 1(c)), although the activation rate was quite low. In low phosphate concentrations, BVR was not activated, consistent with a previous report[1]. By contrast, increasing the amount of sodium phosphate (>100 mM) resulted in activation. In the absence of phosphate ion, the activity of *Syn* BVR in was 17.08 nmol min^{-1} per mg of protein, increasing 2.5-fold to 42.40 nmol min^{-1} per mg of protein in 350 mM phosphate. By contrast, the activity of human BVR increases ~40-fold in the presence of phosphate; thus, phosphate has a relatively minor effect on the activity of *Syn* BVR. In the structure of the apo-*Syn* BVR, phosphate ion derived from sample buffer (PBS buffer) is visible (Supplementary Fig. 1(d)). This inorganic phosphate ion binds to the same site as the 2'-phosphate group of NADP(H) (Supplementary Fig. 1(d)). Therefore, the bound inorganic phosphate probably helps NAD(H) to bind if it were NADP(H), as reported previously for human BVR previously[6].

Supplementary Note 2. Enzymatic properties of the H84A and D287A mutants of *Syn* BVR

Prof. T. J. Mantle's group has performed sophisticated kinetic experiments on BVRs, and found that the H84A and D285A mutations of *Syn* BVR dramatically reduced the enzyme's activity[1]. These residues, however, engage in no direct interaction with bound $NADP^+$ or bound BV (Supplementary Fig. 4(a)). We investigated why these mutations decrease activity.

In the case of the H84A mutant, we observed white precipitates formed during purification. Therefore, we assessed the stability of the supernatant using a thermal-shift assay. The denaturing curves (thermogram) for wild-type and R185A-mutated BVRs were very similar (Supplementary Fig. 4(b)): the estimated T_m values were ~64°C for apo-form and 70°C in the presence of $NADP^+$. By contrast, the thermogram of the H84A mutant clearly indicated denaturing behavior even at room temperature, consistent with circular dichroism (CD) analysis of this mutant[1]. This result strongly suggests that H84 near the active pocket plays a crucial role in stabilizing the folding of this protein.

In the case of D285A (hereafter D287A, because in *Synechocystis* sp. PCC 6803, position 285 is arginine, not aspartate; under our experimental conditions, the D287A mutation dramatically reduced the activity, as previously reported). The D287A mutant had a thermogram similar to those of wild-type BVR and the R185A mutant. However, the thermogram for the apo-form implied that D287 confers greater stability. Interestingly, the titration curve indicated that the D287A mutant can interact with only one biliverdin molecule (Supplementary Fig. 4(c)), and we believe that this explains the dysfunction of the mutant. According to reported K_m values[1], NADPH binds tightly to this mutant: the K_m value for NADPH is 10-fold lower for D287A than for wild-type BVR. In addition, we observed that structural changes occurred upon NADP(H) binding, allowing two biliverdin molecules to be bound, and this structural flexibility is required for BVR activity. Asp287 is probably responsible for fine-tuning of the position of His129, because Asp287 forms hydrogen-bond networks among Arg25 (α1), His126 (β5), Tyr302, and His129 (Supplemental Fig. 4(a)). His129 is involved in the structural change upon NADP(H) binding, as described in the main text. However, we believe that the function of the Asp278 is limited in cyanobacterial BVR because Asp278, like Arg25 and Tyr302, is not conserved among mammalian BVRs (Supplementary Fig. 1).

Supplementary References

1. Hayes, J. M. & Mantle, T. J. The effect of pH on the initial rate kinetics of the dimeric biliverdin-IXα reductase from the cyanobacterium *Synechocystis* PCC6803. *FEBS J.* **276**, 4414-4425 (2009).
2. Hirota, S. *et al.* Cytochrome *c* polymerization by successive domain swapping at the

C-terminal helix. *Proc. Natl. Acad. Sci. USA* **107**, 12854-12859 (2010).

3. Hayashi, Y. *et al.* Domain swapping oligomerization of thermostable c-type cytochrome in *E. coli* cells. *Sci. Rep.* **6**, 19334 (2016).

4. Kravets, A., Hu, Z., Miralem, T., Torno, M. D. & Maines, M. D. Biliverdin reductase, a novel regulator for induction of activating transcription factor-2 and heme oxygenase-1. *J. Biol. Chem.* **279**, 19916-19923 (2004).

5. Lerner-Marmarosh, N. *et al.* Human biliverdin reductase: a member of the insulin receptor substrate family with serine/threonine/tyrosine kinase activity. *Proc. Natl. Acad. Sci. USA* **102**, 7109-7114 (2005).

6. Franklin, E. *et al.* Activation of biliverdin-IXα reductase by inorganic phosphate and related anions. *Biochem. J.* **405**, 61-67 (2007).

7. Thompson, J. D., Higgins, D. G. & Gibson, T. J. CLUSTAL W: improving the sensitivity of progressive multiple sequence alignment through sequence weighting, position-specific gap penalties and weight matrix choice. *Nucleic Acids Res.* **22**, 4673-4680 (1994).

8. Robert, X. & Gouet, P. Deciphering key features in protein structures with the new ENDscript server. *Nucleic Acids Res.* **42**, W320-324 (2014).

Supplementary Table 1 Oligonucleotides used in this study

Oligonucleotide	Sequence
***Synechocystis* sp. PCC 6803**	
BVR-Y102F-F	5’-TTTCCTTTAGCGTTAACCTATGC-3’
BVR-Y102F-R	5’-TTCCAACACCACATGCCCAC-3’
BVR-T169A-F	5’-GCCTATCACCATCAGCAATTTG-3’
BVR-T169A-R	5’-CCAACGTTGGGGAGCGGG-3’
BVR-S184A-F	5’-GCGCGCATCAGTCGGTTTACGG-3’
BVR-S184A-R	5’-CAAGGCCGCCACTAAAGGA-3’
BVR-R185A-F	5’-GCCATCAGTCGGTTTACGGATTT-3’
BVR-R185K-F	5’-AAAATCAGTCGGTTTACGGATTTAT-3’
BVR-R185-R	5’-GGACAAGGCCGCCACTAAA-3’
BVR-R188A-F	5’-GCGTTTACGGATTTATTCGGTACA-3’
BVR-R188A-R	5’-ACTGATGCGGGACAAGGCC-3’
BVR-K237A-F	5’-GCAGGGGAAGTTTTTCACCAGAA-3’
BVR-K237A-R	5’-GCCATAGATAACCTCCGCTT-3’
BVR-R246A-F	5’-GCGATTTTTACCCTCCATGGCG-3’
BVR-R246A-R	5’-TTCATTCTGGTGAAAAACTTCC-3’
**Human**	
BVR-Y98F-F	5’-TTTCCCATGACACTGTCTCTTGC-3’
BVR-Y98F-R	5’-TTCGACCAACACGTGTTTGC-3’
BVR-R172A-F	5’-GCGTTAACCTGGCTTGTCTCCTT-3’
BVR-R172E-F	5’-GAATTAACCTGGCTTGTCTCCTT-3’
BVR-R172K-F	5’-AAATTAACCTGGCTTGTCTCCTT-3’
BVR-R172-R	5’-GCTAATGCCGGAAAACGCA-3’
**Rat**	
BVR-Y97F-F	5’-TTTCCCATGACACTGTCATTTGC-3’
BVR-Y97F-R	5’-TTCCACGAGGACATGCTTG-3’
BVR-R171A-F	5’-GCGCTGACCTGGCTGGTCTCC-3’
BVR-R171E-F	5’-GAACTGACCTGGCTGGTCTCC-3’
BVR-R171K-F	5’-AAACTGACCTGGCTGGTCTCC-3’
BVR-R171-R	5’-AGAAATGCCGCTGAACGCAG-3’

Underlines indicate the altered codons for site-directed mutagenesis.

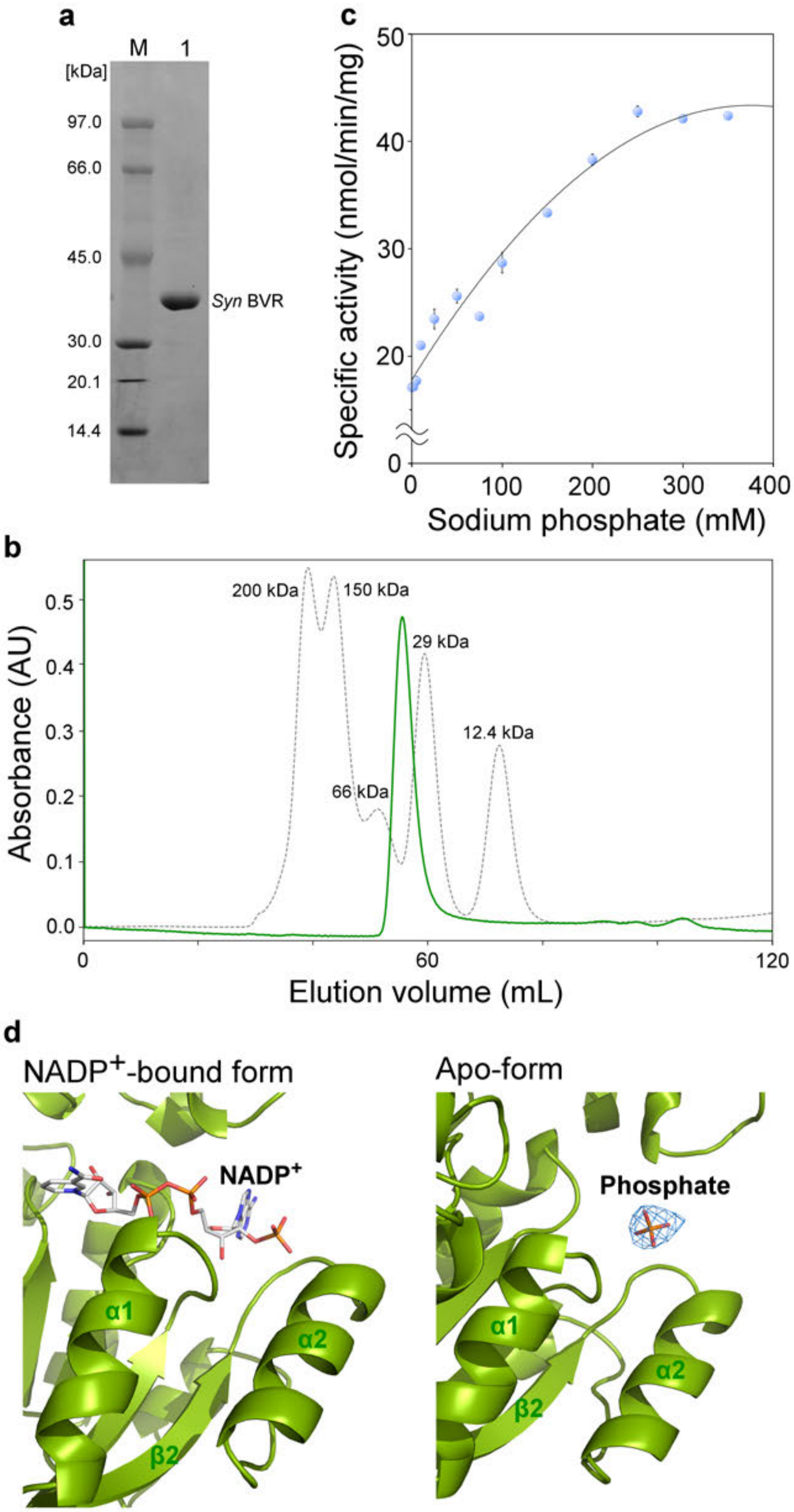

Supplementary Figure 1. Characterization of *Syn* BVR. **(a)** SDS–PAGE analysis of purified *Syn* BVR. Lane M, molecular weight markers (kDa); lane 1, purified non-tagged *Syn* BVR. The gel was stained with Coomassie blue. **(b)** Elution profiles from the gel-filtration column (Sephacryl S-200) are shown with those of size marker proteins, monitored by absorbance at 280 nm. **(c)** Effect of phosphate on activity of *Syn* BVR. n = 3 for each assay. **(d)** Binding site of the phosphate in $NADP^+$ bound- and apo- *Syn* BVR. An omit F_o–F_c map for inorganic phosphate contoured at 3.0 σ (cyan) is overlaid on stick models in the apo-form (*right*).

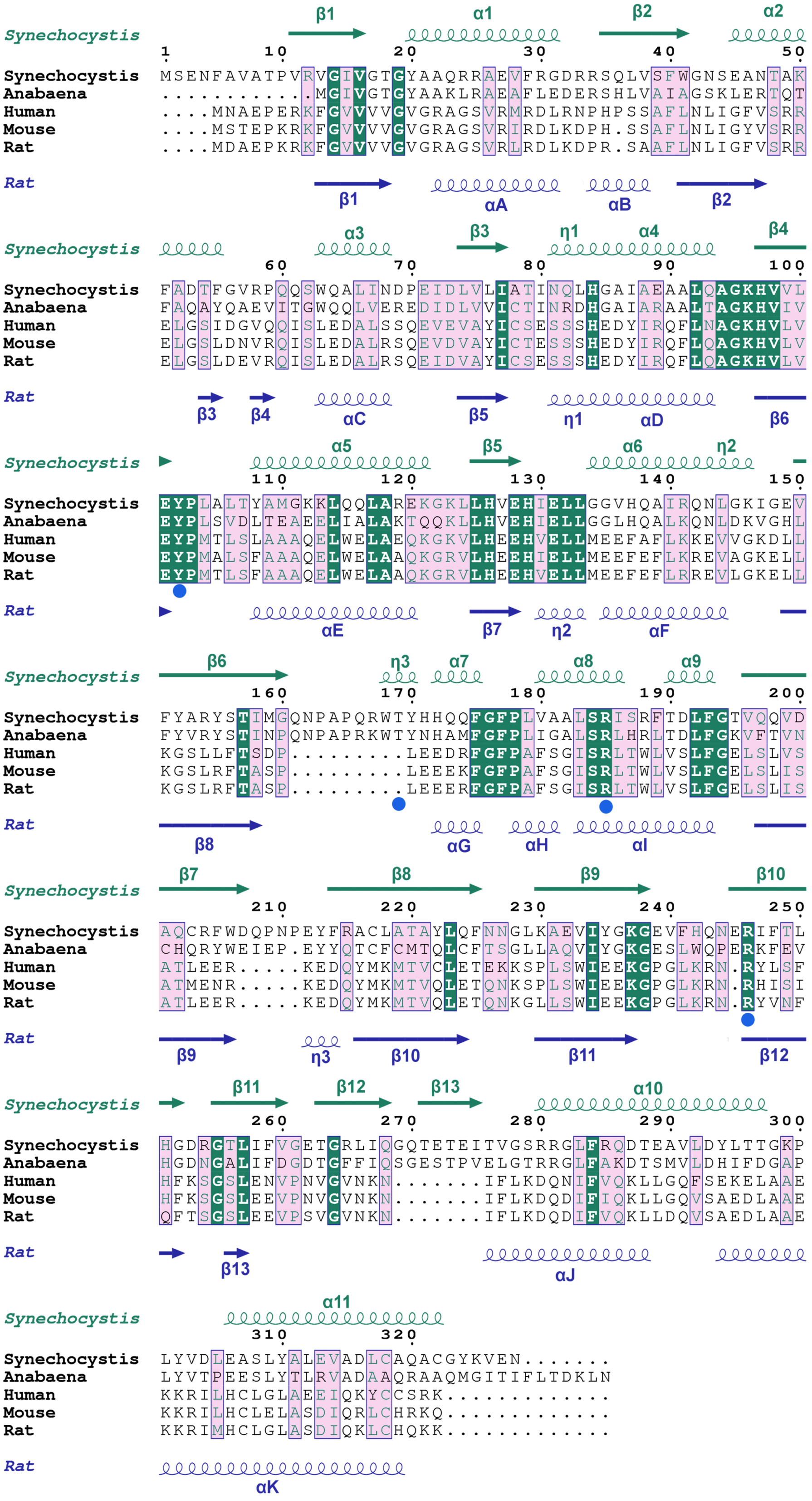

Supplementary Figure 2. Multiple sequence alignment of BVRs from several representative organisms. Sequence numbering is shown for *Syn* BVR. Identical residues are highlighted in green, and similar residues are boxed in pink. The residues that hydrogen bond with BV are indicated by blue filled circles below the alignment. The secondary structural elements of *Syn* BVR and rat BVR are shown above and below the alignment, respectively, and 3_{10} helices are labeled η. The figure was prepared with ClustalW[7] and ESPript 3.0[8]. Synechocystis, *Synechocystis* sp. PCC 6803 (UniProt accession no. P72782); Anabaena, *Anabaena* sp. 7120 (Q8YPP4); human, *Homo sapiens* (P53004); mouse, *Mus musculus* (Q9CY64); rat, *Rattus norvegicus* (P46844).

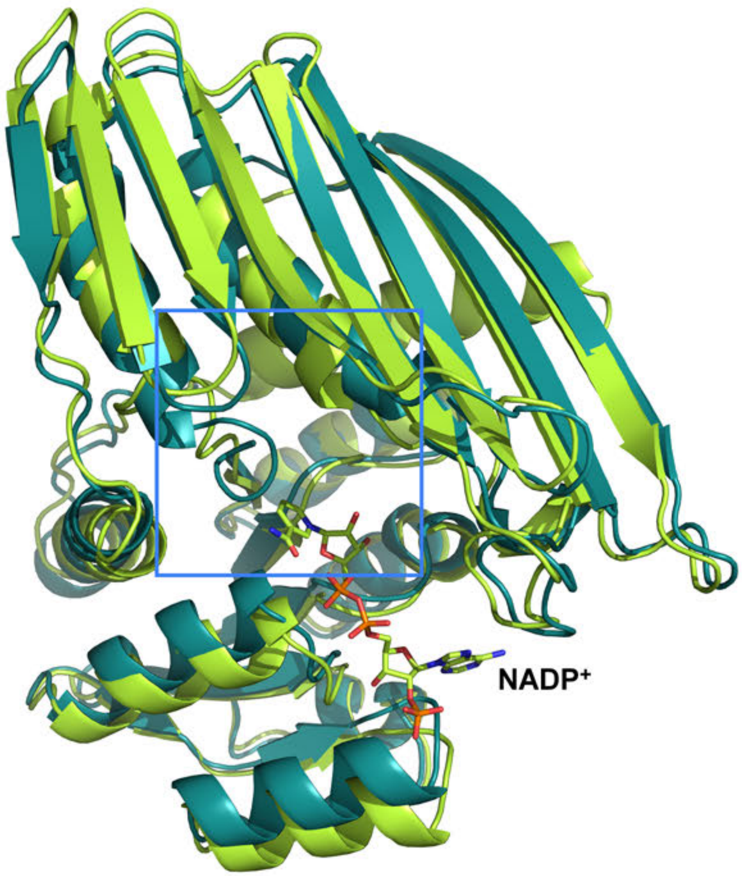

Supplementary Figure 3. Structural rearrangements of *Syn* BVR upon $NADP^+$ binding. The structures of the apo-form (teal green) and $NADP^+$-bound form (light green) are superposed. The close-up view in the square box is represented in Fig. 3(b) in the main text to provide details of the structural rearrangements.

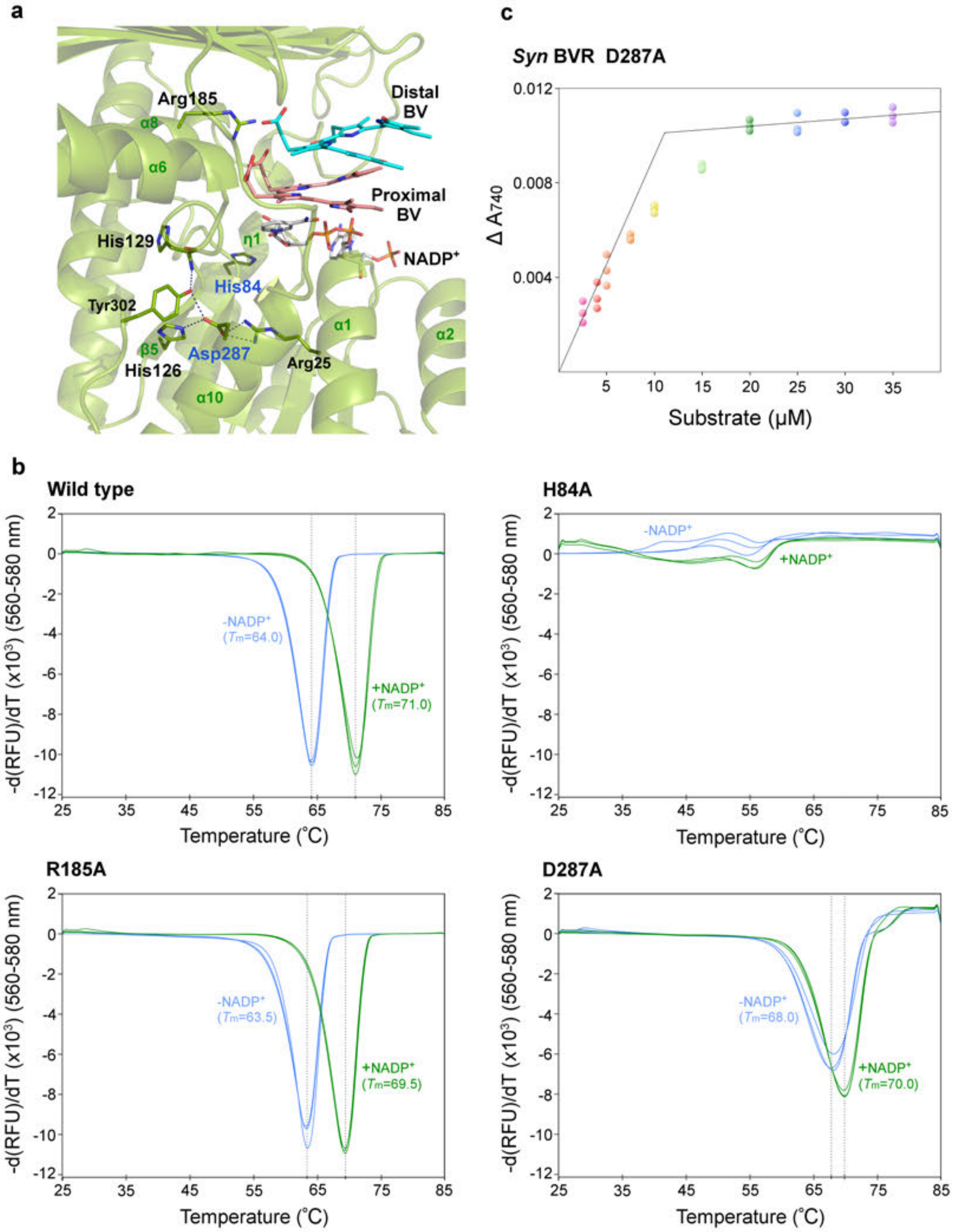

Supplementary Figure 4. Characterization of the H84A- and D287A-mutated *Syn* BVRs. **(a)** Locations of His84 and Asp287 residues in the BV-complex of *Syn* BVR. **(b)** Thermal-shift assays of wild type and the R185A, H84A, and D287A mutants. Inverse first-derivative plots in presence (green) or absence (blue) of $NADP^+$. Average T_m values are shown alongside each curve. n = 3 for each assay. **(c)** Titration curve of D287A-mutated *Syn* BVR (10 µM). Measurements were performed in triplicate.

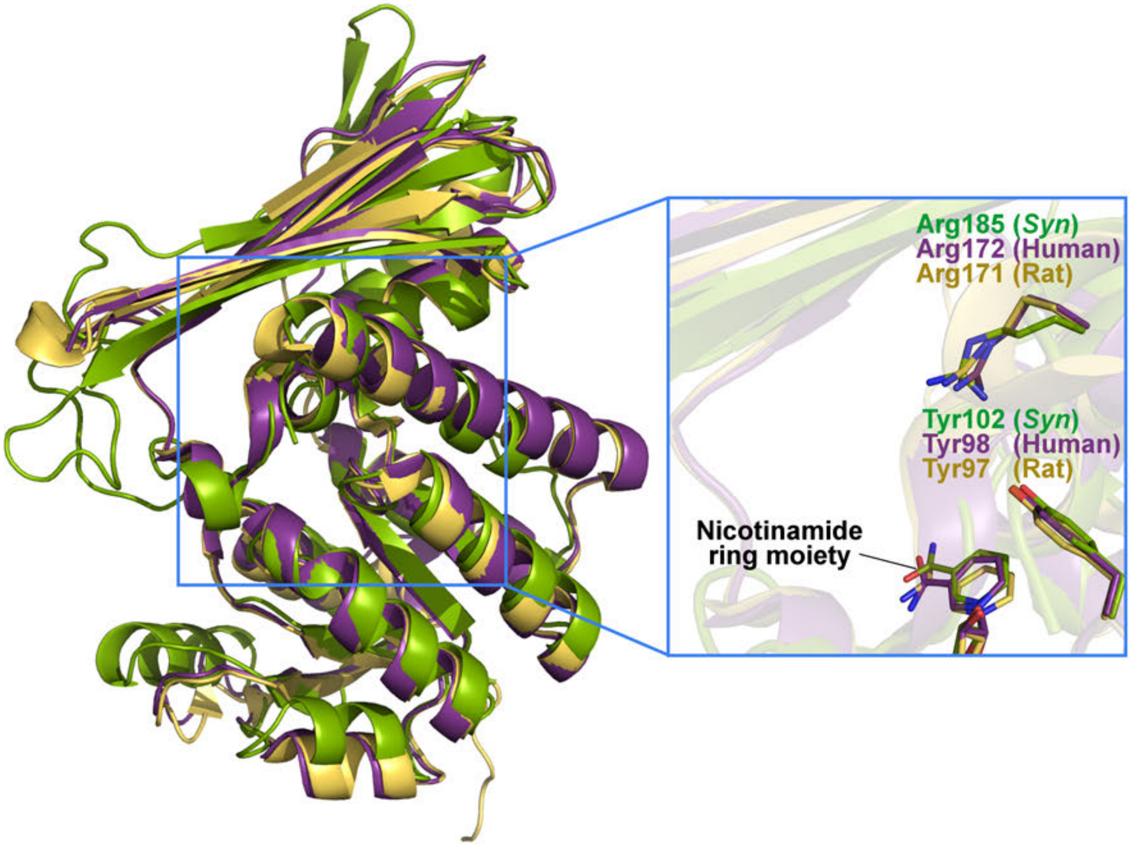

Supplementary Figure 5. Structural comparison of *Syn*, human, and rat BVRs. *Left*, Superimposition of the overall structure of *Syn* BVR (green, *Syn* BVR-NADP$^+$-BV complex), human BVR (purple, NADP$^+$-bound form [PDB ID: 2H63]), and rat BVR (yellow, NAD$^+$-bound form [PDB ID: 1LC3]). The bound BV in *Syn* BVR is omitted to clarify the configuration of the surrounding residues. The BV binding pocket is indicated in the square box, and the magnified view corresponding to this region is represented in the *right* panel.

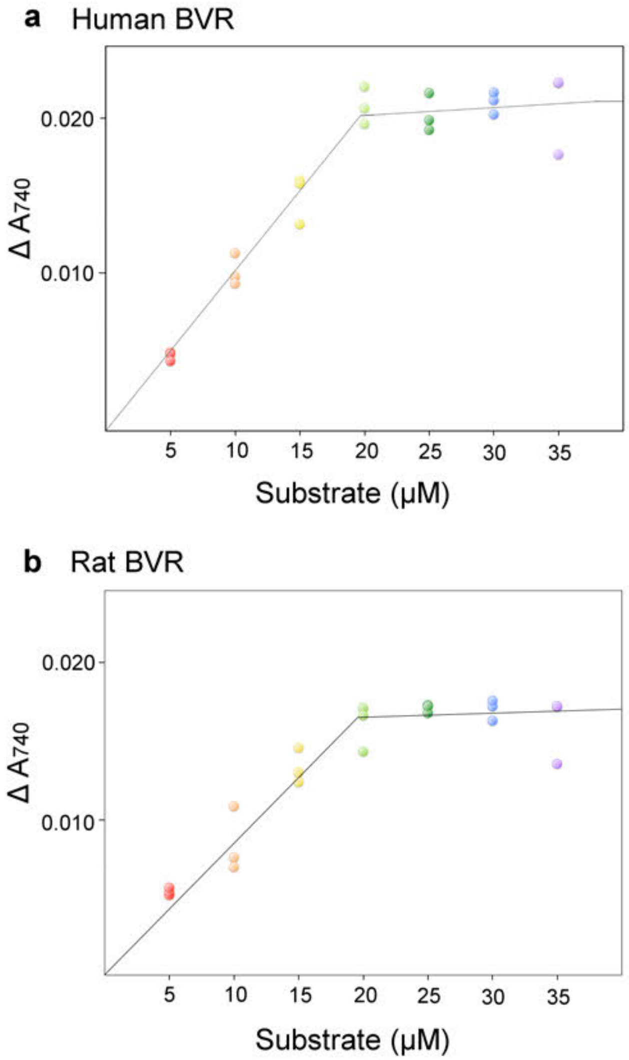

Supplementary Figure 6. Stoichiometry of BV binding to human and rat BVRs. **(a)** Titration curve of human BVR (10 μM) observed at 740 nm. **(b)** Titration curve of rat BVR (10 μM). Measurements were performed in triplicate.
