# Supplementary material for: A substrate-bound structure of cyanobacterial biliverdin reductase identifies stacked substrates as critical for activity
Source: Nat Commun. 2017 Feb 7;8:14397. doi: 10.1038/ncomms14397 (PMC5309722; doi:10.1038/ncomms14397)
Supplement: Peer Review Article [file ncomms14397-s2.pdf]

PEER REVIEW FILE

Reviewers' comments:

Reviewer #1 (Remarks to the Author):

This paper entitled: "A substrate-bound structure of cyanobacterial biliverdin reductase identifies stacked substrates as critical for activity" provides the first structure of Biliverdin reductase with the biliverdin (BV) substrate bound. This structure has been long sought after for many years. It is nice to see it finally determined! The structure also reveals an unexpected binding of substrates, namely two BV molecules bind stacked, one on top of the other. Additionally, using structure-guided site-directed mutagenesis and enzyme kinetics, the authors identify a key catalytic residue, Arg185, which is totally conserved. The structure presented, together with past biochemical and newly presented biochemical data result in the authors proposing a unique catalytic mechanism, where the propionate group of one BV serves as a proton "conduit" to transfer to the other BV molecule situated above the NADPH. This mechanism is consistent with past experimental data that show the BV substrate must have a propionate group to be reduced.

Overall the paper is well and concisely written. The structures are of high quality with acceptable statistics, although the R-free is a little high in the NADP+ complex (8.8% higher than R-work). The conclusions drawn by the authors is consistent with the data presented and the proper controls were adequately used. The findings of this manuscript are appropriate for the journal and should be published after addressing some concerns outline below.

Concerns:
1.) The authors talk about the conformation changes of Arg185 between the apo-and the NADP+ bound structure. Could this "flexible" Arg185 directly transfer protons to distal propionate group? (in proposed mechanism).
2.) In Table 1, give the I/sigma(I) for the highest resolution shell.
3.) Line 77, they list eight beta strands of the C-terminal domain as numbers 1-8, which should be 6-13, to be consistent with Supplementary Figure 1 and previous structures.
4.) Re-word sentence in lines 120-122. They state "two salt bridges" one with Arg188 the other with Tyr102, I think they mean Arg185.
5.) Re-word sentence in lines 141-143. It is confusing. "structure revealed residues that interact are not present". How can a structure reveal something that is not present? I think they mean: Structure reveals that only Tyr102 interacts with C10...
6.) In lines 162-163 the authors talk about the "identical" Arg185 dispositions among Syn, human and rat BVRs. Which conformation of Arg185 are they referring to since two structures reveal two different conformations.

7.) Line 195, remove extremely of “extremely rapidly”
8.) Lines 212-213 talking about propionate side chain of distal BV approaching C10, but their proposed mechanism donates proton to B-ring pyrrole nitrogen (not C10). What is this distance?
9.) Lines 53 & 227 the authors refer to a “dissociation of terminal carboxy group.” This sounds like the carboxy group dissociates off the BV. I think they mean a “carboxyl group with a dissociable proton.”
10.) Figure 3b, the color for the apo structure is listed as “dark green” but it looks more teal than dark green. (Sup Fig.
11.) Finally, given that it appears BV can’t bind alone, only binds with small amounts of contaminating NADP+ (base on their crystal structure), and the reported substrate inhibition (based on enzyme kinetics), suggests a sequential ordered binding of substrates and release of products. Maybe after BV is reduced to BR, BR comes off the enzyme, but under high BV concentrations, a new BV can bind to enzyme before oxidized NADP+ releases, thus trapping an inhibited complex. i.e., NADPH binds first followed by BV, then BR releases, followed by NADP+. Is there reported kinetic data that supports this?

Reviewer #2 (Remarks to the Author):

This is the first structure of a BVR-A with biliverdin in the active site. Not one but two molecules are clearly seen! The authors make much of an sdm study that shows reduced activity with various mutations at R185 and propose a complicated mechanism to deliver a proton to C10 or the nitrogen of pyrrole ring B. There is also discussion that this may assist in the design of inhibitors for the human enzyme to treat neonatal jaundice. It should be noted that similar impairments in kcat have been reported previously for the D285A mutant with even lower activity for the H84A mutant [FEBS J. (2009) 276, 4414-4425].

Unfortunately the authors have not stressed that the cyanobacterial enzyme is distinct in many ways from all of the mammalian forms that have been described to date. The synBVR-A is clearly a dimer [FEBS J. (2009) 276, 4414-4425], where all other forms described to date are monomers. It exhibits a very narrow, acid, pH optimum [JBC (1997) 272, 13562-13569] in contrast to the mammalian enzyme which exhibits activity over a broad pH range [Biochem. J. (2007) 405, 61-67]. It is also not activated by phosphate and other anions, with NADH as the nucleotide substrate, a phenomenon seen with the mammalian forms [Biochem. J. (2007) 405, 61-67]. The physiological role played by synBVR-A is far from clear as it apparently competes (not very effectively) with the ferredoxin-dependent bilin reductases which produce the light harvesting pigments of the phycobilisomes.

That being said the observation that the cyanobacterial enzyme can clearly bind two molecules of biliverdin is an intriguing observation! The authors do not comment on some of the kinetic implications of this observation. The initial rate kinetics might be expected to show some deviation from hyperbolic behavior if two molecules of biliverdin are required for activity (unless there is a product liberation step after binding of the first molecule). The authors also make no reference to QM/MM studies on human BVR-A [J.Phys.Chem. B (2012) 116, 9580-9594) which are consistent with protonation of the pyrrole nitrogen before hydride transfer (this is also seen with the mechanistically related BVR-B, which clearly binds one molecule of biliverdin [Biochem. J. (2008) 411, 475-484]. In both these QM/MM studies the proton donor is suggested to be water.

It would be nice to see the spectrophotometric titration of the rat and human forms of BVR-A to ascertain whether these too show evidence for two molecules of biliverdin binding per subunit. This is a fairly crucial experiment!

Replies to reviewer #1:

Reviewer #1 commented that "The structures are of high quality with acceptable statistics, although the R-free in is a little high in the $NADP^+$ complex (8.8% higher than R-work)." According to this suggestion, we further refined all structures by applying tighter restraints (X-ray/geometry weight, secondary structure restraint, dihedral angle restraint [X-ray/ADP optimization). These refinements improved the R_{free} values of the structures. We changed the values in Table 1 accordingly, and replaced the coordinate files deposited in PDB.

1) Reviewer #1 asked, "Could this flexible Arg185 directly transfer protons to distal propionate group?" Our structural analyses revealed significant structural rearrangements: Given that Arg185 moved to the BV-binding site upon $NADP^+$ binding, the side chain of Arg185 could

form a hydrogen bond to the propionate group of the proximal BV. Although Arg185 is also located near the propionate side chain of the distal BV (~3.1 Å), a hydrogen bond may not be formed due to the parallel configuration between the amino acid residue and the side chain. That is, Arg185 enables direct transfer of the proton to the propionate side chain of the distal BV, but an additional small structural rearrangement is needed. (Lines 222-224 in the main text).

2) We added I/Sigma(I) for the highest-resolution shell in Table 1. We found mistakes in the statistics for the Au-derivative data in Table 1, and corrected them.

3) Reviewer #1 pointed out, "The authors should correct the numbers of the beta-strand in the C-terminal domain, to be consistent with Supplementary Figure 1 and previous structures." Accordingly, we corrected the numbering of the beta-strands (Line 79 in the main text).

Reviewer #1 pointed out ambiguous sentences in comments 4) to 10), and also kindly provided adequate revisions for most of them. Accordingly, we corrected the sentences as follows:

4) In lines 120–122 in the original version, "two salt bridges, one with Arg188 the other with Tyr102, I think the authors mean Arg185." We corrected the sentence accordingly (Lines 122-123 in the main text).
5) Re-word sentence in lines 141–143. "The structure revealed residues that interact are not present. It is confusing". According to the reviewer's suggestion, we modified the sentence to "The structure reveals that only Tyr102 interacts with C10." (Lines 144–145 in the main text)
6) In lines 162–163 in original version, "which conformation of Arg185 are they referring to since two structures reveal two different conformations?" We added a detailed description to clarify the important structural similarity (Lines 167–169 in the main text).
7) According to the reviewer's suggestion, we removed "extremely" from "extremely rapidly" on line 207 in the main text.
8) The reviewer asked, "What is this distance between the propionate side chain of distal BV and B-ring pyrrole nitrogen?" We added the possible distances between them only by rotation of the propionate side chain (line 226).

9) We corrected “dissociation of terminal carboxy group” to “carboxyl group with a dissociable proton” (line 240 in the main text).

10) In the legend of Figure 3b, we changed the name of the color (dark green→teal green).

11) Reviewer #1 asked, “NADPH binds first followed by BV, then BR releases, followed by NADP+. Is there reported kinetic data that supports this?” To date, Prof. Timothy J. Mantle’s group has performed sophisticated kinetic analysis of *Syn* BVR, and found that cyanobacterial BVR obeys an ordered steady-state kinetic mechanism, with NADPH the first to bind and NADP+ the last to dissociate [J.M. Haynes and T.J. Mantle, FEBS J. (2009) 276, 4414-4425]. In our experiments, NADP(H) binding was the trigger for biliverdin binding, consistent with the previous report. We added sentences to address this point (lines 241–244 in the main text).

Replies to reviewer #2:

<u>Comment in 1st paragraph</u>: It should be noted that similar impairments in kcat have been reported previously for the D285A mutant with even lower activity for the H84A mutant [FEBS J. (2009) 276, 4414-4425].

We believe that Prof. Timothy J. Mantle’s group has reported elegant work on BVRs. The discussion of *Syn* BVR in the *FEBS J* article was based on these sophisticated kinetic experiments. To respond to the reviewer’s comments, we further investigated why these mutations dramatically decrease enzyme activity by creating and analyzing the corresponding mutant proteins. In the revised manuscript, we added descriptions and figures about the H84A and D285A (which we believe corresponds to the D287A mutant) (lines 147–150 in the main text, Supplementary Notes, and Supplementary Fig. 4). Details are follows.

In the case of the H84A mutant, we observed white precipitates formed during purification. Therefore, we assessed the stability of the supernatant using a thermal-shift assay. The results revealed that the H84A mutant was denatured even a room temperature (Supplementary Fig. 4(b)), consistent with a CD (circular dichroism) analysis of this mutant

(FEBS J. (2009) 276, 4414-4425). This result strongly suggested that H84 near the active pocket plays a crucial role in stabilizing the folding of this protein

In case of D285A, reported in the *FEBS J* article, the situation is more complicated. First of all, in the amino acid sequence of BVR in *Synechocystis* sp. PCC 6803, position 285 is an arginine residue, not an aspartate. According to the predicted structure of *Syn* BVR in the *FEBS J* paper (Figure 7), D285 residue might interact with H126. Therefore, we believe that the D287A mutant of *Syn* BVR corresponds to the original D285A mutation. Accordingly, we further investigate various parameters using the D287A mutant. Indeed, under our experimental conditions, D287A mutant protein decreased the activity, as reported in *FEBS J*.

The thermogram (thermal melting curve) of the D287A mutant is similar to those of wild-type BVR and the R185A mutant (Supplementary Fig. 4(b)). However, the thermogram implied that D287 conferred greater stability in the apo form. At the very least, mutation in Asp287 position did not affect protein stability/folding as in the case of the H84A mutant. Interestingly, the titration curve indicated that D287A mutant can interact with only one biliverdin molecule (Supplementary Fig. 4(c)), explaining the dysfunction of D287A mutant. According to the K_m values reported in *FEBS J*, NADPH bound tightly to the mutant (Table 2), and the K_m value for NADPH of the mutant is 10-fold lower than that of wild-type BVR. We found that structural changes occur upon NADP(H) binding, allowing two biliverdin molecules could be bound, and this structural flexibility was required for BVR activity. Asp287 is probably responsible for fine-tuning of His129 because Asp287 forms hydrogen-bond networks among Arg25 (α1), His126 (β5), Tyr302, and His129 (Supplementary Fig. 4(a)) in *Syn* BVR. His129 is involved in this structural change, as already described in the original manuscript (lines 90–102). However, we believe that the function of Asp278 is limited to cyanobacterial BVR, as Asp278 (as well as Arg25 and Tyr302) is not conserved among mammalian BVRs (Supplementary Fig. 1).

<u>Comment in 2nd paragraph</u>: the cyanobacterial enzyme is distinct in many ways from all of the mammalian forms that have been described to date.

As the reviewer points out, the previous study reported that *Syn* BVR forms a dimer [FEBS J. (2009) 276, 4414-4425], whereas all other forms described to date are monomers. In the previous report, *Syn* BVR was expressed as a fusion protein with GST, and heat treatment under extreme conditions (6 M urea at 95°C for 2 min) was applied during its purification. In our study, purified *Syn* BVR was obtained by a completely different purification procedure. Under our experimental conditions, *Syn* BVR clearly exists as a monomer, as determined by size-exclusion chromatography (Supplementary Fig. 1(b)) and dynamic light scattering (lines 2–27 in Supplementary Notes), similar to human and rat BVRs. Monomeric *Syn* BVR was not tolerant to heat treatment (6 M urea at 95°C for 2 min), which resulted in denaturation and formation of a heavy white precipitate.

Although the exact reason for the differences in the pH profile of enzymatic activity among mammalian and cyanobacterial BVRs remains uncertain, it may be due to local variations in the amino acids involved in NADP(H) binding and/or the structural changes associated with BV binding. Regarding the enhancement of NADH-dependent activity by phosphate, we observed similar enhancement in *Syn* BVR activity by inorganic phosphate, although the activation rate was very low. When sample buffer was exchanged with Tris-HCl buffer by dialysis, we observed enhancement of the NADH-dependent activity at high concentrations of phosphate (lines 28–41 in Supplementary Notes, and Supplementary Fig. 1(c)). In this measurement, great care was taken to control pH, because the enzymatic activity of *Syn* BVR is highly pH-sensitive. In the apo-*Syn* BVR structure, a phosphate ion derived from the PBS sample buffer is visible. This inorganic phosphate ion binds to the same site as 2’-phosphate group of NADP(H) (Supplementary Fig. 1(d)). Therefore, the bound inorganic phosphate probably helps NAD(H) bind as if it were NADP(H).

Together, these data suggested that the enzymatic properties of *Syn* BVR are reasonably similar to those of mammalian BVRs. The physiological function for BVR in cyanobacteria remains still controversial, but we believe that the enzymatic reaction mechanisms of *Syn* BVR are good model for those of mammalian BVR.

Comments in 3rd paragraph: The initial rate kinetics might be expected to show some deviation from hyperbolic behavior if two molecules of biliverdin are required for activity. The authors also make no reference to QM/MM studies on human BVR-A.

We agree with the reviewer's comment. The initial rate kinetics might reflect the two substrate binding steps. In response to this comment, we attempted to measure the initial rate using stopped-flow equipment. However, we could not observe any deviation from hyperbolic behavior. We believe that the biliverdin binding followed by the structural change upon NADPH binding is faster than we expected, and/or that the binding rate for each biliverdin molecule is comparable. To the best of our knowledge, the initial rate of pig spleen BVR exhibited sigmoidal behavior [M. Noguchi, et. al., *J. Biochem*. 86, 833-848].

The reviewer also mentioned previous QM/MM studies. We added descriptions of these previous reports (lines 249–253 in main text), and we anticipate that further QM/MM studies will be performed based on our structure.

Final comment in last paragraph: It would be nice to see the spectrophotometric titration of the rat and human forms of BVR-A to ascertain whether these too show evidence for two molecules of biliverdin binding per subunit. This is a fairly crucial experiment!

We greatly appreciate this helpful comment. Accordingly, we added titration curves of human and rat BVRs (Supplementary Fig. 6) and relevant description (lines 183–186 in the main text).

Other alterations

To respond to the reviewer's comments, we performed the titration experiments of rat and human BVRs (and initial rate measurements using a stopped-flow spectrometer). Accordingly, we added two authors because they contributed to the measurements and the discussions.

REVIEWERS' COMMENTS:

Reviewer #1 (Remarks to the Author):

The authors satisfactorily addressed all the concerns raised in the initial review process. I feel the article is ready to be published.

Reviewer #2 (Remarks to the Author):

The revised manuscript represents extensive attention to the comments of the reviewers and now reads very well. Only one point in the Supplementary notes requires attention. Lines 17/18 states that "SynBVR expressed in E.coli apparently forms dimers when heat treatment (6M urea at 95 degrees C for 2 min.) is applied during purification" and cites ref 1. In ref 1 this treatment was ONLY used "prior to HPLC analysis" to permit analysis of bound pyridine nucleotides. The discrepancy between the present paper and Ref1 on the oligomeric nature of synBVR-A will need to be addressed elsewhere.

Reviewer's comments:

Reviewer #1 (Remarks to the Author): The authors satisfactorily addressed all the concerns raised in the initial review process. I feel the article is ready to be published.

Reviewer #2 (Remarks to the Author): The revised manuscript represents extensive attention to the comments of the reviewers and now reads very well. Only one point in the Supplementary notes requires attention. Lines 17/18 states that "SynBVR expressed in *E.coli* apparently forms dimers when heat treatment (6M urea at 95 degrees C for 2 min.) is applied during purification" and cites ref 1. In ref 1 this treatment was ONLY used "prior to HPLC analysis" to permit analysis of bound pyridine nucleotides. The discrepancy between the present paper and Ref1 on the oligomeric nature of *syn* BVR-A will need to be addressed elsewhere.

Replies to reviewer #2:

Reviewer #2 pointed out, "Lines 17/18 in the Supplementary notes states that "SynBVR expressed in E.coli apparently forms dimers when heat treatment (6 M urea at 95 degrees C for 2 min.) is applied during purification" and cites ref 1. In ref 1 this treatment was ONLY used "prior to HPLC analysis" to permit analysis of bound pyridine nucleotides."

As in the reviewer's comment, we have been misunderstanding about the purification steps of Syn BVR in the previously reported paper [J.M. Haynes and T.J. Mantle, FEBS J. (2009) 276, 4414-4425]. Therefore, we deleted the sentence of "when heat treatment (6 M urea at 95°C for 2 min.) is applied during purification" in the Supplementary notes. The possible explanations about the oligomeric states (the monomeric and dimeric Syn BVR) remain unchanged (Lines 17-26 in Supplementary notes), because we believe that the monomeric and dimeric states reflect physiological functions of Syn BVR.
